# β-Cell Autophagy Pathway and Endoplasmic Reticulum Stress Regulating-Role of Liposomal Curcumin in Experimental Diabetes Mellitus: A Molecular and Morphometric Study

**DOI:** 10.3390/antiox11122400

**Published:** 2022-12-02

**Authors:** Safaa I. Khater, Mohamed F. Dowidar, Aya E. Abdel-Aziz, Tarek Khamis, Naief Dahran, Leena S. Alqahtani, Mohamed M. M. Metwally, Al-Sayed Al-Hady Abd-Elrahamn, Mohammed Alsieni, Manal E. Alosaimi, Maram H. Abduljabbar, Amany Abdel-Rahman Mohamed

**Affiliations:** 1 Department of Biochemistry, Zagazig University, Zagazig 44511, Egypt; 2 Department of Pharmacology, Faculty of Veterinary Medicine, Zagazig University, Zagazig 44519, Egypt; 3 Laboratory of Biotechnology, Faculty of Veterinary Medicine, Zagazig University, Zagazig 44519, Egypt; 4 Department of Anatomy, Faculty of Medicine, University of Jeddah, Jeddah 23218, Saudi Arabia; 5 Department of Biochemistry, College of Science, University of Jeddah, Jeddah 80203, Saudi Arabia; 6 Department of Pathology, Faculty of Veterinary Medicine, Zagazig University, Zagazig 44511, Egypt; 7 Department of Human Anatomy and Embryology, Port Said University, Port Said 42511, Egypt; 8 Department of Pharmacology, Faculty of Medicine, King Abdulaziz University, Jeddah 21589, Saudi Arabia; 9 Department of Basic Science, College of Medicine, Princess Nourah bint Abdulrahman University, Riyadh 11671, Saudi Arabia; 10 Department of Pharmacology and Toxicology, College of Pharmacy, Taif University, Taif 21944, Saudi Arabia; 11 Department of Forensic Medicine and Toxicology, Faculty of Veterinary Medicine, Zagazig University, Zagazig 44511, Egypt

**Keywords:** autophagy, ER stress, miRNA, pancreatic β-cells, immunohistochemical reactivity

## Abstract

Background: Autophagy can confer protection to pancreatic β-cells from the harmful effects of metabolic stress by delaying apoptosis. Curcumin (CUR) alleviates oxidative and endoplasmic reticulum (ER) stress, activates autophagy, reduces inflammation, and decreases β-cell damage in type I diabetes. Liposomal CUR (LPs-CUR) has a higher therapeutic value and better pharmacokinetics than CUR. Objectives: We determined LPs-CUR’s ability to alleviate stress, reduce β-cell damage and unraveled the mechanism underlying its protective effect using a streptozotocin (STZ)-induced type I diabetic rat model. Methods: *Sprague–Dawley* rats were grouped into vehicle control, STZ-diabetic (STZ 65 mg/kg), STZ-diabetic-3-MA (3-methyladenine [3-MA] 10 mg/kg b.wt), STZ. diabetic-LPs-CUR (LPs-CUR 10 mg/kg b.wt), and STZ diabetic-LPs-CUR-3-MA (LPs-CUR 10 mg/kg b.wt; 3-MA 10 mg/kg b.wt). Results: LPs-CUR significantly reduced blood glucose, oxidative stress, and cellular inflammation in the pancreatic tissue (*p* < 0.001). ER stress-dependent genes included ATF-6, eIF-2, CHOP, JNK, BiP, and XBP LPs-CUR significantly suppressed fold changes, while it upregulated the autophagic markers Beclin-1 and LC3-II. Conclusions: LP-CUR ameliorates β-cell damage by targeting the autophagy pathway with the regulatory miRNAs miR-137 and miR-29b, which functionally abrogates ER stress in β-cells. This study presents a new therapeutic target for managing type I diabetes using miR-137 and miR-29b.

## 1. Introduction

Autophagy delays apoptosis in pancreatic β-cells and induces adaptive responses to alleviate the harmful effects of endoplasmic reticulum (ER) stress and DNA damage, which have been directly linked to oxidative stress [1]. Therefore, it is critical to understand the regulation of cell autophagy in type I diabetes mellitus (DM) for developing new strategies specifically targeting β-cell protection and survival. Nonenzymatic, enzymatic, and mitochondrial mechanisms contribute to oxidative stress in DM [2]. Oxidative glucose metabolism is the main source of nonenzymatic oxidative stress. Reactive oxygen species (ROS) production can be directly influenced by hyperglycemia. Glucose autoxidation can lead to the production of OH radicals [3]. Hyperglycemia and insulin deficiency may be linked to streptozotocin (STZ)-induced selective pancreatic β-cell damage, which is primarily due to DNA alkylation and, to a lesser extent, ROS formation and nitric oxide production [4]. Autophagy has been implicated to have numerous roles, one of which is to aid in proper β-cell function [5]. Under stress conditions, autophagy contributes to cell survival; insulin homeostasis regulation; and damage repair resulting from nutrient deficiency, oxidative stress, endothelial cell stress, mitochondrial damage, and hypoxia [1,6]. Long-term exposure to ROS due to hyperglycemia can damage cells, decrease insulin release in response to glucose, and potentially, result in cell death [7]. The ROS-driven oxidative stress that occurs because of elevated blood glucose levels during the course of diabetes pathogenesis can aggravate the death of β-cells.

In physiological settings, unfolded or misfolded proteins trigger the protective effect of autophagy in mammals experiencing ER stress. Autophagy, in turn, reduces ER stress and delays apoptosis [8]. Protein kinase R-like ER kinase (PERK), inositol-requiring protein 1 (IRE1), and activating transcription factor 6 (ATF-6) are some of the transmembrane ER resident proteins regulating the unfolded protein response (UPR) at its closest point to the E.R. Beclin-1, mTOR, AKT, and ATG8 are some of the genes regulated by these three signaling pathways [9]. Furthermore, both the UPR signaling system and autophagy are simultaneously involved in the ER process [10]. Unfolded or misfolded protein buildup, inflammation, and calcium disorders all lead to autophagy, which clears misfolded proteins and decreases ER stress to prevent cell death, a process called ER stress-induced autophagy [8]. JUN N-terminal kinase (JNK) has been linked to IRE1 activation, which can, in turn, lead to Beclin-1-induced autophagy [11]. PERK-eIF2 is important for autophagy induction after ER stress, which confirms the link between autophagy and the UPR signaling system [12].

The increase in proinsulin and insulin production is attributed to the small interfering RNA-mediated suppression of atg5/7 in β-cell autophagy [5]. Insulin production from mouse and human islets is reduced by the rapamycin-mediated suppression of mTORC1 [13]. Balanced control of autophagy is further underscored by the findings that activation and inhibition of autophagy through knockout or hyperactivation of mTORC1, respectively, resulted in an increase in cell apoptosis and a decrease in β-cell mass when autophagy was activated or inhibited [13,14]. There is accumulating evidence suggesting that autophagy is essential for cell viability and survival, which occurs through various mechanisms.

Type I DM patients account for approximately 10% of the total diabetes population, and its incidence is growing. Type I DM patients require lifelong insulin injections for their survival [15]. This disease occurs because of inflammatory infiltration of the islets of Langerhans (insulitis) and selective destruction of insulin-producing β-cells [16]. DM patients increasingly seek safer medications that have antidiabetic activity and reduced risk of severe side effects [17,18].

To preserve β-cells’ morphological and functional integrity, antioxidant therapy, which limits free radical generation from damaged cells, may be necessary. Several antioxidant therapies are available and are seen as promising new treatment options [19]. Curcumin (CUR) has the potential to be used as a therapeutic or nutraceutical agent because of its ability to target multiple pathological disorders. CUR can help maintain good health in ailments such as metabolic syndrome, arthritis, anxiety, and hyperlipidemia, all of which are associated with oxidative and inflammatory stress [20]. Adrenergic receptors and critical cell signaling molecules can be regulated by CUR pretreatment, which improves insulin gene expression and insulin production [21]. However, the limitations of CUR as a therapeutic agent are minimal stability, low water solubility, ineffective penetration, poor bioavailability, rapid metabolization and elimination, and low targeting efficacy [22]. Since the 1960s [23], liposomes have been used as carrier molecules in drug delivery. They are useful for application in several fields ranging from pharmaceuticals to cosmetics, and they can be used to transport a wide variety of nanomaterials. Phospholipids and steroids make up most of these particles, which have a size range of 50–450 nm. Liposomal CUR (LPs-CUR) treatment is more powerful and effective than CUR solution treatment, with significant improvements in the function of the liver and pancreatic indicators and oxidative/antioxidative parameters in STZ-diabetic rats.

To confirm the mechanistic pathway of LPs-CUR, we hypothesized that the effect of this compound would be inhibited by the administration of an autophagy inhibitor (3-methyl adenine [3-MA]), which was confirmed by comparing the degree of expression of the genes and the immunohistochemical staining reaction of Beclin-1 and LC3 proteins. To test this hypothesis, the present study aimed to assess whether the administration of LPs-CUR would be helpful in alleviating ER stress and activating autophagy, which would consequently restore glucose and insulin levels and reduce β-cell damage. Additionally, the mechanism underlying this protective effect was unraveled in the current study by estimating the expression pattern of genes related to ER stress and the autophagy pathway and their epigenetic regulation by microRNA in the β-cells of an STZ-induced type I DM rat model.

## 2. Materials and Methods

### 2.1. Animals and Test Compounds

At Zagazig University’s Veterinary Animal House, we procured fifty nine-week-old male Sprague–Dawley rats (weighing 250 g). They have 15 days to get used to the new environment. The Zagazig University-Institutional Animal Care and Use Committees ZU-IACUC committee of Zagazig University, Egypt, ensured that all rats were cared for according to the standard procedures. The ZU-IACUC/2/F/24/2022 card identifies the user.

Sigma-Aldrich Chemical Co. provided the STZ Powder (St. Louis, MO, USA). A chemical termed 3-methyladenine (3-MA, an autophagy inhibitor) was procured from Sigma-Aldrich and delivered in powder form, and distilled water was used to dissolve the powder. When needed, the powder was dissolved in 0.1 mol/L cold citrate buffer, Ph 4.45, and stored at –20 °C.

### 2.2. Experimental Animals

Fifty *Sprague–Dawley* male rats (9 weeks old; 280 ± 5 g) were obtained from the laboratory animals farm, Faculty of Veterinary Medicine, Zagazig University. All rats were held in cages of stainless steel maintained in an atmosphere free of pathogens at 21–24 °C, 60% relative humidity, and a 12 h light–dark cycle. The rats got ad libitum filtered water and fed regular rodent chow. Before starting the experiment, the rats were allowed to acclimatize for two weeks. All research procedures were complied with the NIH recommendations for the Care and Use of Laboratory Animals and approved by the Ethics of Animal Use in Research Committee (IACUC), Zagazig University, Egypt, with the reference number (ZU-IACUC/2/F/24/2022).

### 2.3. Preparation and Characterization of LPs-CUR

The ethanol injection approach was used to prepare curcumin liposomes, as it had previously been [24,25]. After dissolving the organic phase in 5 mL of 100% ethanol, HSPC (76.2 mg), cholesterol (12.8), and curcumin (20 mg) were stored at 60–70 °C. To prepare the aqueous phase, sucrose (900 mg) was dissolved in 10 mL of deionized water and held at 60–70 °C while stirring (700 rpm). Using a 23G syringe, we introduced the heated organic phase into the pre-prepared aqueous phase. Excess ethanol was removed from the mixture by heating it to 60 degrees Celsius and stirring it for 20 min [26]. For the liposomal suspension stability, it was kept at 4 °C. The average particle size, polydispersity index [PDI], and zeta potential of the obtained liposome solution were measured by dynamic scattering light analysis using Malvern Nano-ZS.

### 2.4. Induction of T1DM

After a fasting state that lasted overnight, STZ. (65 mg/kg, dissolved in 0.1 M cold citrate buffer, Ph 4.5) was administered intraperitoneally (IP) to induce type I diabetes in rats. STZ-injected rats got sucrose (15 g/L) in their water for 48 h to prevent early death from insulin release from partially damaged pancreatic islets. Those rats with fasting blood glucose levels greater than 250 mg/Dl were included in the experiment after 72 h. Subcutaneous injection of long-acting insulin (2–4 U/rat) was used to keep blood glucose levels in diabetic rats within a range (350 mg/Dl) and prevent ketonuria from developing whenever it is needed [27].

### 2.5. Experimental Design

The experiment was conducted over ten weeks, in which the experimental rats were randomly divided into five groups (10 rats per group). As the following

Group I (vehicle control): During the diabetes induction, rats were given 1 mL/kg b.wt of citrate buffer via oral gavage and distilled water for the rest of the study time.

Groups II (STZ-diabetic): the rats received a single IP of STZ. (65 mg/kg b.wt. dissolved in 0.1 M citrate buffer saline) [27].

Group III (STZ-3MA): Once a day for three weeks (at the end of experimental periods) (from the 8th week to the end of the experiment), STZ-diabetic rats received IP of 3-MA (10 mg/kg b.wt) into their abdomens [28].

Group IV (STZ-LPs-CUR): STZ-diabetic rats were administered liposomal curcumin (LPs-CUR) orally at a dose level of (10 mg/kg b.wt.) for ten weeks.

Group V (STZ-LPs-CUR-3MA): Liposomal-curcumin (LPs-CUR) was administered orally to STZ-diabetic rats at a dose level of (10 mg/kg b.wt.) for 10 weeks and injected intraperitoneally with 3-MA (10 mg/kg), once per day, for the last three weeks only (from the 8th week to the end of the experiment).

### 2.6. Dose Selection Strategy of LPs-CUR

LPs-CUR was given to rats in this selected dose (10 mg/kg b.wt.) according to the results previously obtained by Adriana et al. [29] when the authors were comparing the efficacy of CUR and LPs-CUR to alleviate the complications in experimentally induced diabetes Mellitus, and the results indicated the better efficacy of LPs-CUR as compared to curcumin solution on all serum levels of liver enzymes, oxidative stress, and the markers of pancreatic damage.

### 2.7. Sample Collection

Sodium pentobarbital (I/P, 100 mg/kg) was used to anesthetize the rats after ten weeks to reduce suffering and distress for the animals. Blood samples were obtained from the medial canthus of all rats in tubes without an anticoagulant. Three portions of a dissected and homogenized rat pancreas were immediately collected, frozen, and stored at –80 °C for total RNA extraction, histological and immunohistochemical examinations, and assays of oxidant/antioxidant activity. Finally, the rats were weighed and starved overnight before being euthanized in an airtight container with 4 percent fluothane in oxygen.

### 2.8. Biochemical Examinations

#### 2.8.1. Blood Glucose Level, β-Cell Function, Insulin-Resistant, and Insulin Sensitivity

Fasting blood glucose was estimated by the glucose oxidase method (Biodiagnostic, Giza, Egypt). The β-cell function, insulin sensitivity, and insulin resistance were calculated using HOMA calculators (Diabetes Trials Unit, 2019).

#### 2.8.2. Serum Insulin and C-Peptide

The insulin level (ng/mL) was estimated by the commercial ELISA technique of Biovender R&D (Catalog. No. RA19004R), and C-Peptide (ng/mL) was measured by an ELISA kit (CUSABIO, Catalog. No. CSB-E05067r).

#### 2.8.3. Pancreatic Oxidants/Antioxidants Status

Pancreatic levels of Malondialdehyde MDA were measured by an ELISA kit from MyBioSource (San Diego, CA, USA, cat no. MBS8807536). Superoxide dismutase (SOD.) (CAT.NO A1957), glutathione peroxidase GPX (CAT.NO A2883), and Catalase (CAT) (CAT.NO A4590) were estimated using ELISA Kits from Antibodies.com Egypt.

#### 2.8.4. Pancreatic Pro-Inflammatory Cytokines

The levels of tumor necrosis factor-α (TNF-α), Interferon Gamma (IFNg) interleukin-6 (IL-6), and interleukin-10 (IL-10) were estimated in the serum of each group according to the manufacture instructions illustrated in ELISA each kit procedure, produced by Thermofisher scientific Co, (Waltham, Massachusetts, United States) Catalog # KRC3012, Cat #ERIFNG, Cat #ERA31RB, and Cat #ERA23RB, respectively.

### 2.9. Real-Time RT-PCR

Total RNA extraction was performed as previously described [30]. For mRNA, 500 ng of that total RNA was reverse-transcribed as previously described [4,31]. In contrast, for miRNA, 10 ng were reverse-transcribed using the TaqMan™ Small RNA Assays (ThermoFisher Scientific, Waltham, MA, USA) following manufacturer instructions. The Stem-loop RT and miR-specific primers and the universal reverse primer were designed using the (http://genomics.dote.hu:8080/mirnadesigntool, accessed on 10 September 2020) assay design software [32]. Primers were manufactured by Sangon Biotech (Beijing, China) and presented in Table 1. Thermo Fisher Scientific, Waltham, MA, USA, provided the Maxima SYBR Green/Rox qPCR Master Mix (2X) for real-time PCR. A 2CT was used to calculate the relative expression of each gene normalized to either housekeeping B-actin or U6 (for mRNA and miRNA, respectively) [33].

### 2.10. Histopathological Examination

All the dead animals during the experiment and the live animals at the end of the experimental period were necropsied following a standardized necropsy protocol [34]. The whole left lobes of the pancreas of all animals were collected following the guides for organ sampling and trimming in rats [35]. The pancreatic tissues were cut longitudinally in a horizontal plane to make the cut surface as large as possible and fixed in a 10% neutral buffered formalin solution for 24 h. The tissue specimens were processed post-fixation for the paraffin technique [36]. Briefly, the specimens were thoroughly washed in distilled water, dehydrated in ascending series of ethyl alcohol, cleared in Histo-Clear II (Scientific Laboratory Supplies Ltd., Nottingham NG11 7EP UK.) clearing agent, processed for paraffin impregnation, and embedding, sectioned at five µm thick, stained with Harris hematoxylin and eosin, and examined microscopically. The histological changes in the exocrine or endocrine tissues were recorded, and quantitative lesion scoring and morphometric analysis were carried out. Concisely, for each rat, ten randomly selected microscopic fields provided that every single field contains one or more islets of Langerhans were snapshotted. Next, the surface area fractions of the islets were calculated using the Java ima 5 processing program, ImageJ version 1.33, and the frequencies of the recorded histological alterations (congestions, hemorrhages, cellular vacuolations, apoptosis, necrosis, and inflammatory cell infiltrates) were quantified using the formula:

FQ (**%**) = N_lesion_ × N_total_^−1^ × 100

where: (N_lesion_) is the number of images exhibiting a lesion and (N_total_) is the total number of images per group.

### 2.11. Beclin-1 and LC3 Immunohistochemical Investigation

Immunostaining of the Beclin-1 and LC3 autophagy markers was done on the formalin-fixed, paraffin-embedded pancreatic tissue sections using anti-Beclin 1 (ab217179) (Abcam Inc. Waltham, Boston, US), at 1/200 dilution and anti-LC3B antibody [EPR21234] (ab232940) (Abcam Inc.), at 5 µg/mL dilution primary antibodies followed by conjugation to the secondary antibodies and 3,3′-Diaminobenzidine (DAB) staining following the avidin-biotin-peroxidase immunohistochemical technique developed by [37]. Next, the Beclin-1 and LC3 immunoexpression were quantified in twenty randomly selected islets of Langerhans per animal. The percentages of the positively stained Beclin-1 and LC3 brown area fractions concerning the total areas of the islets of Langerhans were calculated using the ImageJ software via the color deconvolution plugins. The results were expressed as percentages (means ± SE) using a six-point area fraction scale as follows: negative expression, 0; weak expression, ≤10%; mild expression, >10%–≤25%; moderate expression, >25%–≤50%; strong expression, >50%–≤75%>; and overexpression, >75%.

### 2.12. Statistical Analysis

Tukey’s Multiple Range test evaluated the results after a one-way analysis of variance (ANOVA). GraphPad INSTAT was used to perform the statistical analysis (Version 2). An acceptable *p*-value was 0.001 or below. Data were also plotted and analyzed using a computer application known as Graphpad (ISI Software, Philadelphia, PA, USA). A Shapiro–Wilk W test was used to determine whether the data were normal, and the significance threshold was set at *p* < 0.05%. The significance which may be significant at * means when *p* < 0.05, ** means when *p* < 0.01, *** means when *p* < 0.001 and **** *p* < 0.0001). 

## 3. Results

### 3.1. Characterization of LPs-CUR

**Figure 1:**
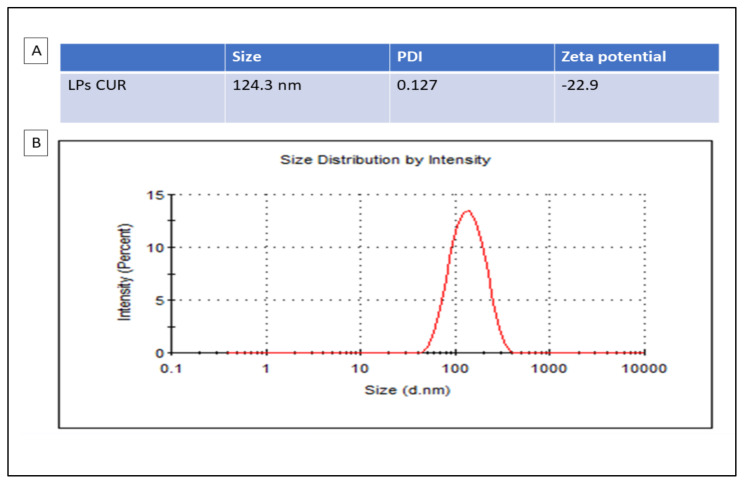
Particle sizes analysis with DLS (dynamic light scattering) are presented in the table (**A**) and the distribution by zeta potential of LPs-CUR is presented in the graph (**B**).

Figure 1 shows the particle size distribution and zeta potential of LPs-CUR.

### 3.2. Glucose, Insulin, and C-Peptide Levels in the Treatment Groups

**Figure 2:**
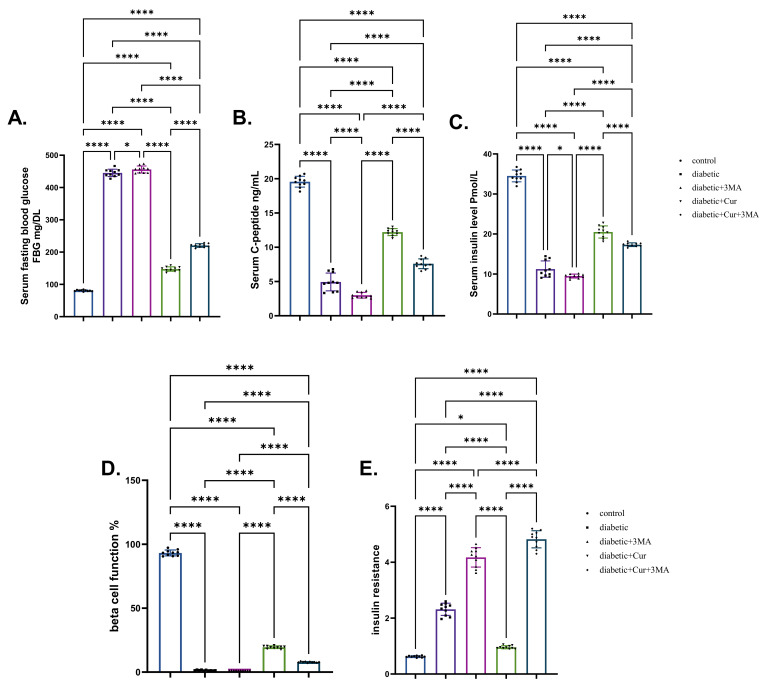
Effect of diabetes (STZ) (65 mg/kg b.wt), 3MA (10 mg/kg b.wt), LPs-CUR (10 mg/kg b.wt.) oral dosing for 10 weeks on (**A**) serum blood glucose, (**B**) C-Peptide, (**C**) Serum insulin, (**D**) Beta cell function %, (**E**) insulin resistance. Data expressed as mean ± SE, *n* = 10 for each group. Each bar carrying the significance which may be significant at * means when *p* < 0.05, and **** *p* < 0.0001).

STZ-diabetic rats showed a significant increase (6.23-fold increase) in the glucose level and a significant decrease in the insulin and C-peptide levels (decrease by 88.51% and 74.53%, respectively) when compared with the vehicle control rats (*p* < 0.001). A substantial increase in glucose levels was seen in the STZ. diabetic-3-MA group when compared with that in the STZ-diabetic group. Additionally, 3-MA reduced insulin levels and C-peptide levels when compared with that in the vehicle control group. LPs-CUR treatment reduced glucose levels in STZ-diabetic rats (by 5.12-fold) while simultaneously increasing insulin and C-peptide levels (by 44.26% and 47.22%, respectively) when compared with those in STZ-diabetic rats. When 3-MA was combined with LPs-CUR, a significant increase in glucose levels and a significant decrease in insulin and C-peptide levels were seen A (*p* < 0.001; Figure 2).

### 3.3. Oxidative Stress and Antioxidants

**Figure 3:**
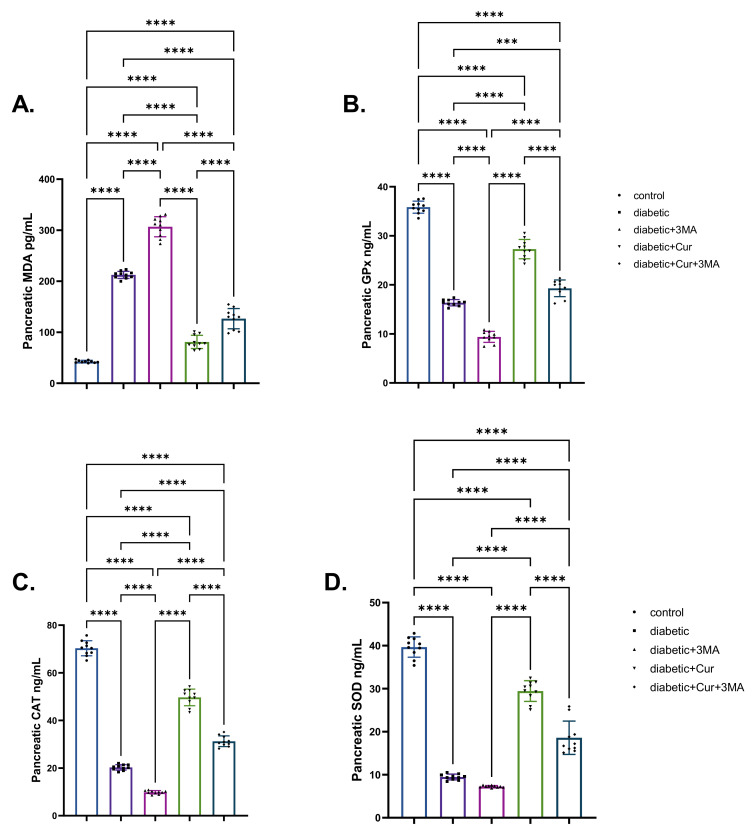
Effect of diabetes (STZ) (65 mg/kg b.wt), 3MA (10 mg/kg b.wt), LPs-CUR (10 mg/kg b.wt.) oral dosing for 10 weeks on (**A**) MDA., (**B**) GPX., (**C**) C.A.T., (**D**) SOD. in pancreatic tissue of all diabetic and vehicle control groups. Data expressed as mean ± SE, *n* = 10 for each group. Each bar carrying the significance which may be significant at *** means when *p* < 0.001 and **** *p* < 0.0001 at *p* < 0.001.

As shown in Figure 3, STZ diabetic rats in all groups had significantly higher levels of pancreatic MDA than control rats (*p* < 0.001). The STZ-diabetic-3-MA group had the highest level of pancreatic MDA, followed by the STZ diabetic group (4.03-fold higher than that in the vehicle control group) and the combination group (STZ. Diabetic-LPs-CUR; 2.04-fold higher than that in the vehicle control group). The STZ-diabetic-LPs-CUR group had the lowest increment (87.91%) when compared with the vehicle control group. Regarding the antioxidant enzymes (SOD, GPX, and CAT), the induction of type I DM caused significant decrements in these enzymes activity in the STZ. diabetic group by 75.69%, 54.4%, and 70.77%, respectively, relative to the vehicle control group. Furthermore, when the diabetic rats received 3-M.A., the decrements were even more pronounced. When comparing the three diabetic groups, LPs-CUR substantially increased the activation of antioxidants in diabetic rats. Moreover, the administration of 3-MA with LPs-CUR reversed the potentiating antioxidant role of LPs-CUR in STZ-diabetic rats.

### 3.4. Pro-Inflammatory Cytokines

**Figure 4:**
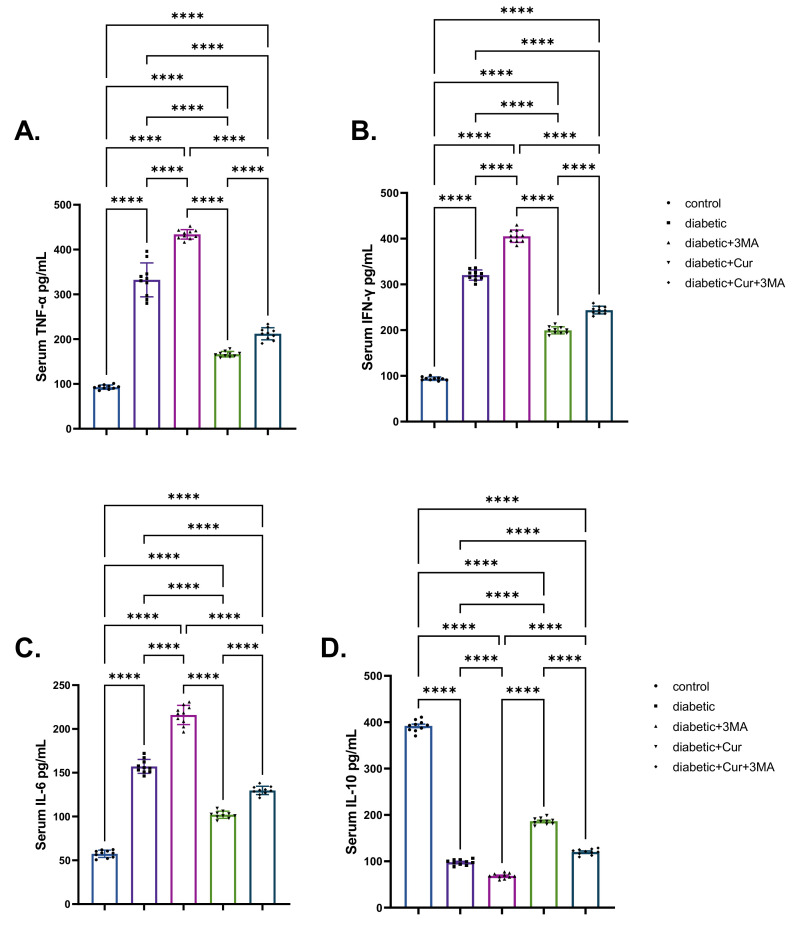
Effect of diabetes (STZ) (65 mg/kg b.wt), 3MA (10 mg/kg b.wt), LPs-CUR (10 mg/kg b.wt.) oral dosing for ten weeks on serum levels of pro-inflammatory cytokines (**A**) TNF-α, (**B**) IFN-Y, (**C**) IL-6, (**D**) IL-10 of all diabetic and vehicle control groups. Data expressed as mean ± SE, *n* = 10 for each group. Each bar carries the significance, which may be significance which may be significant at **** *p* < 0.0001.

When compared with the vehicle control group, the STZ diabetic group showed significant elevation in TNF-α, IFN-γ, and IL-6 levels to different degrees (2.63-fold, 2.42-fold, and 1.71-fold, respectively; *p* < 0.001; Figure 4). By contrast, STZ-induced type I DM STZ significantly reduced the IL-10 level by 75.73% relative to the vehicle control group (non-diabetic group; *p* < 0.001). When compared with the vehicle control group, TNF-α levels were 103% higher, IFN-γ levels were 88% higher, and IL-6 levels were 104% higher in the STZ-diabetic-3-MA group. Importantly, IL-10 levels in the same group were considerably lower by 83.34% than those in the vehicle control group (*p* < 0.001). As shown in Figure 4, the administration of LPs-CUR in STZ-diabetic rats caused a significant reduction in the levels of pro-inflammatory cytokines when compared with those in the STZ-diabetic and STZ diabetic-3-MA groups. However, LPs-CUR elevated the levels of IL-10 when compared with the STZ-diabetic and STZ. diabetic-3-MA groups. By contrast, this effect was reversed when 3-MA was administered throughout the experimental period concurrently with LPs-CUR in STZ-diabetic rats when compared with that in STZ diabetic-LPs-CUR rats.

### 3.5. Effects on mRNA Expression Levels of ER Stress-Dependent and Autophagy-Related Genes in the Pancreatic Tissue

**Figure 5:**
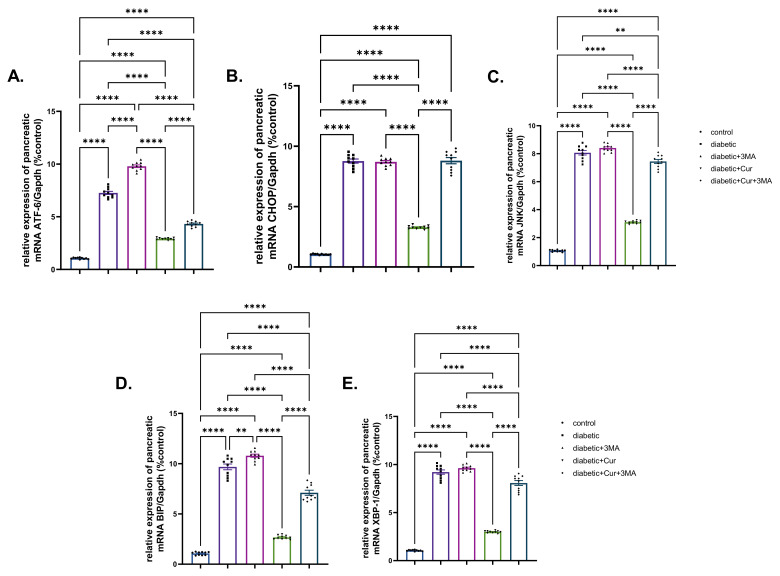
Effect of diabetes (STZ) (65 mg/kg b.wt), 3MA (10 mg/kg b.wt), LPs-CUR (10 mg/kg b.wt.) oral dosing for 10 weeks on the expression pattern (Fold change) of genes by qRT-PCR (**A**) Atf-6, (**B**) CHOP, (**C**) JNK., (**D**) BIP, (**E**) XBP-1 in pancreatic tissue of all diabetic and vehicle control groups. Data expressed as mean ± SE, *n* = 10 for each group. Each bar carrying the significance which may be significance which may be significant at ** means when *p* < 0.01, and **** *p* < 0.0001).

Fold changes of ER stress-dependent genes, namely, ATF-6, CHOP, JNK, BiP, and XBP, are presented in Figure 5. All these genes showed significantly upregulated expression upon induction of type I DM in rats (6-fold, 7.29-fold, 6.62-fold, 8.08-fold, and 7.67-fold for ATF-6, CHOP, JNK, BiP, and XBP., respectively) when compared with those of control non-diabetic rats (*p* < 0.001). Similar upregulation patterns (8.30-fold, 7.28-fold, 1.03-fold, 2.24-fold, and 60%) were observed in the STZ-diabetic-3-MA group relative to those in the diabetic group. By contrast, significant suppression of gene expression (ATF-6, CHOP, BiP, and XBP) was identified, with fold changes of 4.23, 5.34, 4.67, 6.51, and 5.83, respectively, owing to LPs-CUR treatment to STZ diabetic rats when compared with that in the STZ-diabetic group. In the combination group (STZ-diabetic-LPs-CUR 3-MA), a significantly higher increase in the mRNA levels of the detected genes was noted than that in the STZ diabetic-LPs-CUR group.

**Figure 6:**
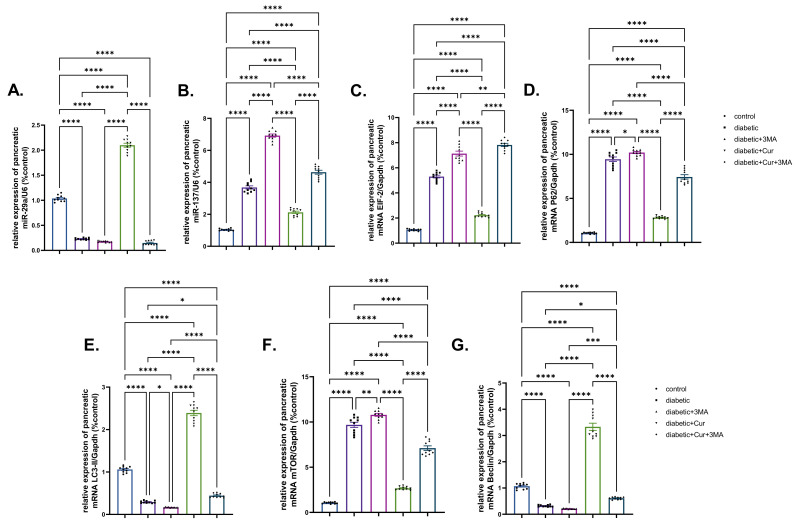
Effect of diabetes (STZ) (65 mg/kg b.wt), 3MA (10 mg/kg b.wt), LPs-CUR (10 mg/kg b.wt.) oral dosing for 10 weeks on the expression pattern (Fold change) of genes by qRT-PCR (**A**) mic-RNA mir-29b, (**B**) mir-137, (**C**) mRNA EIF-2, (**D**) mRNA P62 (**E**) mRNA LC3-II, (**F**) mRNA mTOR, and (**G**) Beclin-1 in pancreatic tissue of all diabetic and vehicle control groups. Data expressed as mean ± SE, *n* = 10 for each group. Each bar carrying the significance which may be significance which may be significant at * means when *p* < 0.05, ** means when *p* < 0.01, *** means when *p* < 0.001 and **** *p* < 0.0001).

Compared with the vehicle control group, miR-29b expression in the pancreatic tissue of rats in the STZ. diabetic group was significantly downregulated by 77.7% (*p* < 0.001; Figure 6). The genes miR-137, eIF-2, and P62 showed strikingly increased expression in the STZ. diabetic group when compared with that in the vehicle control group (2.56-fold, 4-fold, and 7.87-fold, respectively; *p* < 0.001). Moreover, 3-MA treatment to STZ-diabetic rats caused a substantial suppression in miR-29b expression when compared with that in the vehicle control group. It is well known that miRNA 29b and miR-137 expression is associated with autophagy; the obtained results revealed that the mRNA expression of miR-137, eIF-2, and P62 was upregulated in the STZ-diabetic-3-MA group by 5.5-fold, 9.9-fold, and 8.7-fold when compared with that in the STZ-diabetic group. By contrast, the expression of miR-137, eIF-2, and P62 was downregulated by 98.6% in the LPs-CUR group when compared with that in the vehicle control group. Furthermore, the effect of LPs-CUR was significantly dampened when combined with 3-MA.

The expression patterns of LC3-II and Beclin-1 genes were significantly downregulated by 71.61% and 68.75%, respectively, in the pancreatic tissue of the STZ diabetic group (*p* < 0.001; Figure 6E,G) when compared with those in the vehicle control group. Compared with the STZ-diabetic group, 3-MA inhibited LC3II and Beclin-1 expression in the STZ-diabetic-3-MA group. Compared with the vehicle control, mTOR expression was upregulated by 8.08-fold in the STZ-diabetic group and by 1.16-fold in the STZ-diabetic-3-MA group. Moreover, LPs-CUR treatment led to pronounced upregulation of mTOR expression and significantly greater upregulation of both Beclin-1 and LC3-II in the pancreatic tissue of rats in the STZ-diabetic-LPs-CUR group when compared with that in the STZ-diabetic group, STZ-diabetic-3-MA group, and co-treated group (STZ-diabetic-LPs.CUR-3-MA).

### 3.6. Histological Examination Findings

**Figure 7:**
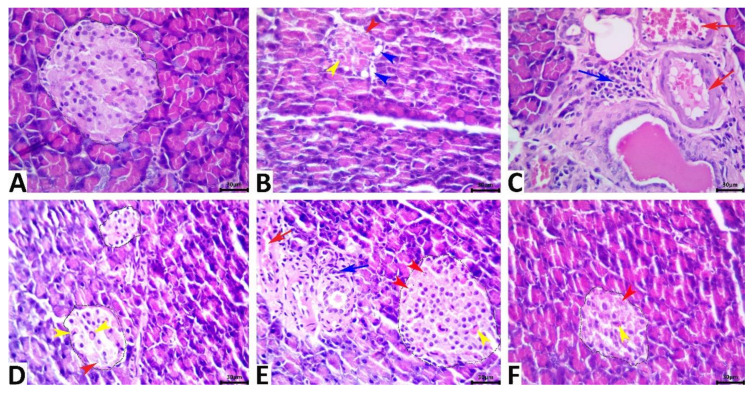
Representative photomicrograph of the hematoxylin and eosin-stained pancreatic tissue sections showing normal histology of the exocrine and endocrine tissues in the vehicle control rat (**A**). The STZ-diabetic pancreas shows a diminished islet of the Langerhans size with β cell vacuolations (blue arrowheads), apoptosis (yellow arrowhead), and necrosis (red arrowhead) (**B**), and periductal mononuclear cell infiltration (blue arrow) with vascular congestion (red arrow) (**C**). The pancreas of the STZ-3MA-treated animal shows β cell apoptosis (red arrowhead), and necrosis (yellow arrowhead) (**D**). The pancreas of the STZ-LPs-CUR-treated animal shows vascular congestion (red arrow), periductal mononuclear cell infiltrate (blue arrow), and β cell apoptosis (yellow arrowhead), pyknosis (blue arrowhead), and chromatorhexis (black arrowhead) (**E**). The pancreas of the STZ-LPs.CUR-3MA-treated animal shows β cell apoptosis (yellow arrowhead), and necrosis (red arrowhead) (**F**). Scale bars = 30 microns.

Microscopic examination findings revealed normal histology in rats of the vehicle control group. Typically, the exocrine tissue of rats is composed of a compound acinar gland, and the endocrine tissue (islets of Langerhans) includes scattered, small aggregates of endocrine cells with diameters ranging from approximately 90 to 200 µm; additionally, the endocrine tissue is composed of closely apposed branching cords of polygonal homogeneous cells surrounded by a basal lamina (Figure 7A). The β-cells constitute most of the islet volume and are located in the center, while the α-cells, δ-cells, and less numerous pancreatic polypeptide cells are located at the periphery. The pancreatic tissue of STZ. Diabetic rats manifested various morphologic alterations characteristic of cell injury; primarily, the alterations in the β cells included β-cell vacuolations, apoptosis, and necrosis, all of which were associated with a remarkable shrinkage in the size of the islets of Langerhans (Figure 7B). Vascular congestion and periductal mononuclear cell infiltration were evident in some of the tissue specimens (Figure 7C). The exocrine tissue elements were almost normal in most of the tissue specimens except for focal atrophic exocrine acini with no fibrotic changes or with inflammatory cell infiltrates in a few tissue specimens. The pancreatic tissue of STZ-diabetic-3-MA rats showed the same histological alterations as those seen in STZ-diabetic rats (Figure 7D). LPs-CUR supplementation showed significant rescue effects against STZ-induced β-cell injury, yet the normal histology of the pancreas could not be regained because most of the pancreatic tissue specimens of rats in the diabetic-LPs-CUR group exhibited a marked reduction in the severity of the β-cell injury, frequent vascular congestion, and periductal mononuclear cell infiltration (Figure 7E). Moreover, the diameter of the islets was greatly restored toward normalcy. Supplementation of 3MA with LPs-CUR significantly diminished the rescue effects of LPs-CUR against STZ-induced β-cell injury because of most of the tissue specimens of rats in the STZ. diabetic-LPs-CUR-3-MA group showed similar but slightly milder lesions to those of rats in the STZ-diabetic group (Figure 7F). Quantitative lesion scoring of pancreatic alterations in all the groups is shown in Table 2.

### 3.7. Immunohistochemical Examination Findings

**Figure 8:**
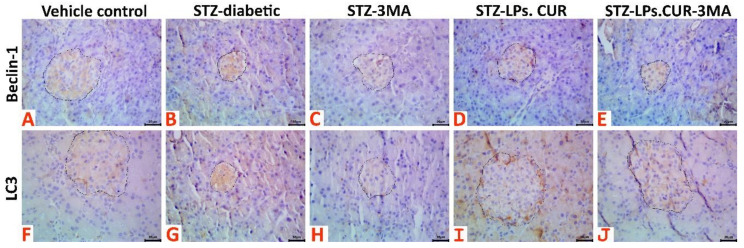
Representative photomicrograph of Beclin-1 stained (**A**–**E**) and LC3 stained (**F**–**J**) pancreatic tissue sections showing upregulation of both biomarkers’ immunoexpression in the STZ-diabetic animals, notable upregulation of both biomarkers in the STZ-LPs-CUR treated animals, and downregulation of both biomarkers’ immunoexpression in the 3MA, and STZ-LPs.CUR-3MA treated animals.

Immunohistochemical analysis (Figure 8) results showed that STZ treatment upregulated the immunoexpression of the autophagic markers, Beclin-1 and LC3, in the islets of Langerhans when compared with those in rats of the vehicle control group. Interestingly, 3-MA treatment downregulated Beclin-1 and LC3 expression when compared with that in rats of the vehicle control group, while LPs-CUR supplementation led to overexpression of both markers. Supplementing 3-MA with LPs-CUR significantly downregulated Beclin-1 and LC3 expression when compared with that in LPs-CUR treatment. Data of the positively stained Beclin-1 and LC3 brown area fractions in 20 islet tissues per animal in all the groups are presented in Table 2.

## 4. Discussion

There is evidence suggesting that elevated plasma glucose levels resulting from insulin resistance play a notable role in DM development [38]. In the first stages of DM, pancreatic cell hypertrophy occurs in response to hyperglycemia and insulin resistance. However, as DM worsens, β-cell dysfunction and death can occur because of the increased metabolic demand. Inflammatory processes, apoptosis, autophagy, and ER stress may be the primary pathways in the etiology and progression of this chronic metabolic disease, despite DM being traditionally known as a nonimmune disorder.

The present study suggests a significant increase in the lipid peroxidation marker MDA and a decrease in antioxidant activity (GPX, SOD, and CAT) in the STZ-diabetic, STZ. diabetic-3-MA, and STZ-diabetic-LPs-CUR-3-MA) groups when compared with those in the diabetic group, a finding that is in accordance with the results obtained by Zhao et al. [39]. The mechanism underlying pancreatic oxidative stress-mediated hyperglycemia is said to occur as follows: First, β-cells are more susceptible to the oxidative environment; they are characterized by excessive production of ROS with a small amount of free radical scavenging system (antioxidant enzyme), which make the β-cells more vulnerable to oxidative stress [5].

Second, hyperglycemia reduces the mitochondrial membrane potential, thereby decreasing energy production and the synthesis of antioxidant enzymes [6].

Third, hyperglycemia shifts glucose fate from normal glycolysis to a ROS-generating pathway [7]. Fourth, glucose autoxidation occurs in addition to β-cell hypoxia and ER stress [5].

However, 3-MA has the potential to inhibit the autophagy pathway, which aggravates β-cells’ negative energy balance and, subsequently, impairs the oxidant scavenging system [8].

Interestingly, the administration of LPs-CUR improved pancreatic oxidative stress, a finding that is in line with the ameliorative effect of LPs-CUR, as reported previously [9,10]. LPs-CUR plays a role in the functional regulation of pancreatic adrenergic receptors, which are G-protein-coupled receptors. The activation of these receptors significantly enhances the phosphorylation of several PI3K/AKT-dependent molecular pathways inhibiting β-cell oxidative stress, associated inflammation, and apoptosis by improving β-cells’ energy balance, thereby strengthening the oxidant scavenging system [11,12,13]. CUR significantly upregulates the expression of α2-adrenergic receptors modulating key signaling molecules that improve insulin gene expression and secretion [11]. Furthermore, CUR directly exerts antioxidant and anti-inflammatory activities [14,15].

In reference to the serum inflammatory cytokine storm associated with STZ-induced type I DM, type I DM significantly (*p* < 0.001) increased serum levels of pro-inflammatory cytokines (TNF-α, IL-6, and INF-ϒ) and decreased the level of the anti-inflammatory cytokine IL-10 (*p* < 0.001), a finding in line with the result previously reported [16,17]. These cytokines participate in the function and differentiation of diabetogenic immune cells [17]. On the same ground, the STZ-diabetic-LPs-CUR-3-MA group showed a significant increase in the serum levels of the pro-inflammatory cytokines, which suggested that switching off autophagy promoted the inflammatory cascades and increased β-cell damage [8,18,19]. However, the aforementioned parameters could reach the normal physiological tone of the control group by the oral administration of LPs-CUR, which strongly proved the anti-inflammatory property of LPs-CUR. LPs-CUR exerts its action through the downstream regulation of NF-ҡβ/ERK/P-ERK, which inhibits the further secretion of other inflammatory cytokines such as IL-6, TNF-α, and INF-ϒ and induces upstream regulation of the PPAR-ϒ signaling pathway that enhances β-cell proliferation [20,21,22]. CUR can prevent cytokine-induced cell death in pancreatic cells by scavenging ROS and regulating cytokine-induced NF-ҡβ translocation (in vitro study) [40].

The inflammatory state of β-cells is aggravated because the UPR is provoked owing to the diabetic β-cells’ negative energy balance being a cellular compensatory mechanism to conserve energy utilization for the vital cellular processes. ER stress signals are disseminated through three conservative pathways, namely, ATF-6, eIF-2 (PERK), and IRE-1 pathways. The main target for the aforementioned pathways is to activate XBP-1 and BiP to inhibit mRNA transcription, wherein the already transcribed mRNA is spliced, and to reduce protein biosynthesis to reduce the cytoplasmic accumulation of unfolded and misfolded proteins, which are considered auto-antigens provoking a subsequent inflammatory response in the form of an autoimmune reaction [23,24,25,26]. The prolonged incapability of the ER and Golgi apparatus of β-cells to carry out post-translational modification of the newly translated proteins leads to overexpression of CHOP and JNK, which finally potentiates inflammation and apoptosis of β-cells [27].

The results of the present study are similar to the aforementioned consequences, as the STZ-diabetic group showed a significant upregulation in the relative expression of the ER stress elements ATF-6, eIF-2, CHOP, JNK, XBP-1, and BiP. The STZ-diabetic-3-MA, and STZ-diabetic groups showed that blocking autophagy leads to further deterioration of the pancreatic energy balance and aggravated UPR, leading to further aggravation of β-cell apoptosis and inflammation [28]. On the contrary, LPs-CUR treatment showed downstream regulation in the expression of the ER stress elements [29]; the ameliorative effect of LPs-CUR could be attributed to its antioxidant and anti-inflammatory activities [20]. Additionally, it improves the β-cell energy balance through functional modulation of β-cell adrenergic receptors [11,12].

Autophagy is the quality control system of cells wherein the damaged organelles are repaired and recycled [30]. It also assists the ubiquitin system in cellular recovery from toxic proteins [31]. Thus, autophagy promotes cell survival and positively regulates energy balance [32]. Prolonged hyperglycemia abrogates the autophagy pathway [33,34,35]. The results of the present study are in accordance with the aforementioned finding, as the gene expression study revealed a significant downregulation in the mean fold change of Beclin-1 and LC3-II and a significant upregulation in mTOR and P62. ER autophagy improves cell survivability, delays apoptosis, and induces adaptive responses to mitigate the detrimental effect of the damaged DNA and ER stress. However, prolonged ER stress, hyperglycemia, and starvation lead to impairment and dysregulation of the autophagy pathway and subsequent cellular damage and death [36,37].

By contrast, the STZ-diabetic-LPs-CUR group showed a significant upregulation in the relative expression of the autophagy genes LC3-II and Beclin-1 by immunohistochemical reactivity and gene expression and a significant downregulation in the expression of p62 and mTOR [38,39]. Inhibition of autophagy in the STZ-diabetic group and the STZ- diabetic-LPs-CUR group worsens or abrogates the therapeutic outcomes of LPs-CUR, as the inhibition-induced functional modulation in the β-cell microenvironment through the potential regulation of autophagy.

Among the autophagy regulatory molecules are microRNAs, which are short noncoding RNA sequences (18–22 nucleotides). They functionally inhibit mRNA translation by targeting the 3′ untranslated regions [40]. miR-137 is an autophagy inhibitor [41]. A recent study showed that miR-137 can increase chemotherapy sensitivity in pancreatic cancer cells by suppressing autophagy mediated by ATG5. miR-137 has the potential to be used as a therapeutic target in the treatment of pancreatic cancer. Our results show that LPs-CUR has the potential to activate and regulate autophagy by decreasing miR-137 expression in the pancreatic tissue of the treated groups when compared with that in the STZ-diabetic and STZ-diabetic-3-MA groups. Consistent with the findings of past research, miR-137 suppressed mitophagy by controlling the expression of the mitophagy receptors FUNDC1 and NIX [41]. Furthermore, the upregulation of miR-137 expression inhibited autophagy in glioma cells [42].

Unlike miR-29b, which upregulated autophagy through the downstream regulation of PI3K/AKT/mTOR pathways, the present study monitored miR-129b expression as an indicator of autophagy activation. The results revealed that the downregulation of mTORC1 expression was due to LPs-CUR exposure in STZ. diabetic rats when compared with that in the STZ-diabetic group. This may be attributed to the ability of LPs-CUR to induce miR-29 overexpression, which led to limited mTORC1 recruitment to lysosomes and inhibition of mTORC1 activity, a finding in line with the result of a recent study [43]. The present study showed significant downregulation in miR-137 expression and significant upregulation in miR-29b expression in the STZ-diabetic-LPs-CUR-3MA group relative to the diabetic control (*p* < 0.001).

## 5. Conclusions

Based on the results of the present study, we conclude that LPs-CUR has the potential to ameliorate STZ-induced β-cell damage by targeting the autophagy pathway with the subsequent regulatory miRNAs (miR-137 and miR-29b), which functionally abrogate β-cell ER stress, oxidative stress, and inflammatory microenvironment. The current study presents a new therapeutic target for the management of type I DM, wherein the main targets are miR-137, miR-29b, and another autophagy inducer. The mechanistic pathway of LPs-CUR was also elucidated in this study by confirming the interrupted action of LPs-CUR when it was concurrently administered with the autophagy inhibitor 3-MA. All of these results indicate the potency of LPs-CUR and provide a greater fundamental understanding of the mechanisms through which LPs-CUR could preserve protein-folding homeostasis and redox status. LPs-CUR can be considered a useful tool to provide new information for the development of novel therapeutics for DM.

## Figures and Tables

**Table 1:** Primer sequences, accession number, and product size for the quantitative RT-PCR for the analyzed genes in the pancreatic tissue.

Gene	Forward primer (5′–3′)	Reverse Primer (5′–3′)	Accession No.	Product Size
**Bclin-1**	GAATGGAGGGGTCTAAGGCG	CTTCCTCCTGGCTCTCTCT	NM_001034117.1	180
**LC-3**	GAAATGGTCACCCCACGAGT	ACACAGTTTTCCCATGCCCA	NM_012823.2	147
**Mtor**	GCAATGGGCACGAGTTTGTT	AGTGTGTTCACCAGGCCAAA	NM_019906.2	94
**P62**	GGAAGCTGAAACATGGGCAC	CCAAGGGTCCACCTGAACAA	NM_181550.2	183
**EIF-2**	CTTTCCGGGACAAGATGGCG	CTCTGTGAAGTGTGGGGGTC	NM_001399818.1	95
**ATF6**	AAGTGAAGAACCATTACTTTATATC	TTTCTGCTGGCTATTTGT	NM_001107196.1	157
**BIP**	AACCAAGGATGCTGGCACTA	ATGACCCGCTGATCAAAGTC	NM_013083.2	240
**CHOP**	CACAAGCACCTCCCAAAG	CCTGCTCCTTCTCCTTCAT	NM_001109986.1	158
**JNK**	AGTGTAGAGTGGATGCATGA	ATGTGCTTCCTGTGGTTTAC	NM_053829.2	182
**XBP1**	TTACGAGAGAAAACTCATGGGC	GGGTCCAACTTGTCCAGAATGC	NM_001004210.2	289

**Table 2:** Effect of diabetes (STZ) (65 mg/kg b.wt), 3MA (10 mg/kg b.wt), LPs-CUR (10 mg/kg b.wt.) oral dosing for 10 weeks on the [pancreatic tissue lesion scoring of all treated groups and control.

Groups	Acinar Atrophy	Size of the Islets of Langerhans in Micrometers	Beclin-1 DAB Area Fraction	LC3 DAB Area Fraction
Control	0.0 b ± 0	130.0 ^a^ ± 8.44	33.52 ^c^ ± 1.86	33.62 ^c^ ± 2.17
Diabetic	12.0 ^b^ ± 2.49	75.8 ^b^ ± 6.47	51.82 ^b^ ± 2.92	47.36 ^b^ ± 1.61
Diabetic + 3MA	31.0 ^a^ ± 6.4	72.2 ^b^ ± 5.95	10.61 ^e^ ± 1.42	12.0 ^e^ ± 1.59
Diabetic + LPs-CUR	1.0 ^b^ ± 1	85.1 ^b^ ± 4.23	77.13 ^a^ ± 0.82	72.9 ^a^ ± 1.16
Diabetic + LPs.CUR-3MA	6.0 ^b^ ± 2.67	80.8 ^b^ ± 3.42	22.08 ^d^ ± 2.21	21.18 ^d^ ± 2

Data expressed as mean ± SE, *n* = 10 for each group. Values carrying different superscripts (a, b, c, d, e) are significantly different.

## Data Availability

All data is contained within the article.
